# TBX1 Represses *Vegfr2* Gene Expression and Enhances the Cardiac Fate of VEGFR2+ Cells

**DOI:** 10.1371/journal.pone.0138525

**Published:** 2015-09-18

**Authors:** Gabriella Lania, Rosa Ferrentino, Antonio Baldini

**Affiliations:** 1 CNR Institute of Genetics and Biophysics Adriano Buzzati Traverso, Via Pietro Castellino 111, 80131 Napoli, Italy; 2 Dept. of Molecular Medicine and Medical Biotechnology, University of Naples Federico II, 80131, Napoli, Italy; University of Houston, UNITED STATES

## Abstract

The T-box transcription factor TBX1 has critical roles in maintaining proliferation and inhibiting differentiation of cardiac progenitor cells of the second heart field (SHF). Haploinsufficiency of the gene that encodes it is a cause of congenital heart disease. Here, we developed an embryonic stem (ES) cell-based model in which *Tbx1* expression can be modulated by tetracycline. Using this model, we found that TBX1 down regulates the expression of VEGFR2, and we confirmed this finding *in vivo* during embryonic development. In addition, we found a *Vegfr2* domain of expression, not previously described, in the posterior SHF and this expression is extended by loss of *Tbx1*. VEGFR2 has been previously described as a marker of a subpopulation of cardiac progenitors. Clonal analysis of ES-derived VEGFR2+ cells indicated that 12.5% of clones expressed three markers of cardiac lineage (cardiomyocyte, smooth muscle and endothelium). However, a pulse of *Tbx1* expression was sufficient to increase the percentage to 20.8%. In addition, the percentage of clones expressing markers of multiple cardiac lineages increased from 41.6% to 79.1% after *Tbx1* pulse. These results suggest that TBX1 plays a role in maintaining a progenitor state in VEGFR2+ cells.

## Introduction

The majority of the cardiac cells derives from two separate populations of progenitors named “heart fields” [1]. In the mouse, the two heart fields are specified during gastrulation [2,3]. The first heart field (FHF) contributes to the left ventricle and part of the atria, while the second heart field (SHF) contributes to the outflow tract (OFT), the right ventricle (RV) and part of the atria [4]. TBX1 is a transcription factor expressed in several tissues but its early expression in mesodermal tissue is particularly important for normal development of SHF-derived heart segments, especially the OFT [5–7]. Mesodermal-specific deletion of *Tbx1* down regulates cell proliferation in the SHF [8] revealing its role in the expansion of cardiac progenitors [9]. In addition, Cre recombination-based cell tracing has shown that descendants of *Tbx1*-expressing cells populate myocardial and endocardial layers of the OFT and RV and, to a lesser extent the right atrium [5,10].

Chen and co-workers [9] showed that *Tbx1* is expressed in and regulates proliferation of multipotent cardiac progenitors. In addition, they showed premature differentiation in the SHF of *Tbx1* ^-/-^ embryos. Furthermore, TBX1 is required for the addition of progenitor cells from the anterior SHF (an anteriorly located sub-population of SHF) to the OFT, and is also important for differentiation at the inflow tract of the heart (IFT) [11]. How TBX1 functions within multipotent cardiac progenitors and how it interacts with other factors important for multipotency are still open questions. Among the factors shown to be expressed in multipotent cardiac progenitors there is VEGFR2, also known as KDR or FLK1 [12]. VEGFR2 is required for hematopoietic and endothelial cell (EC) lineage development [13]. Conditional deletion of the *Vegfr2* gene causes heart defects and loss of endocardial cells [14]. Clonal analysis of embryonic stem (ES) cell-derived VEGFR2^+^ cells showed that they are able to generate cardiomyocyte, vascular smooth muscle and endothelial cells in culture [15]. VEGFR2+ cells isolated from mouse embryos at E7.5 showed a gene expression profile with characteristics of both first and second heart fields [12]. In addition, in vitro differentiation of ES cells produced a population of multipotent cardiac progenitor cells, which expresses *Vegfr2*, *Isl1*, and *Nkx2*.*5* as well as *Tbx1* [16].

Here, we provide evidence that TBX1 is a negative regulator of *Vegfr2* gene and protein expression in differentiating ES cells and *in vivo*. Loss of *Tbx1* in mouse embryos causes expansion of a previously not described VEGFR2 expression domain. Furthermore, analysis of ES-derived clones showed that VEGFR2+ cells are prevalently EC progenitors but, upon induction of *Tbx1* expression, they also expressed marker of cadiomyocyte and smooth muscle lineage. These data suggest that TBX1 supports multipotency of a subpopulation of VEGFR2+ cells.

## Materials and Methods

### Generation of the mES-Tbx1Tet^OFF^ Cell Line and Tissue Culture

Mouse ES EBRTc cells [17] were cultured in DMEM high glucose (Invitrogen Ltd, Paisley, UK, catalogue no. 11995–065) supplemented with 15% fetal bovine serum (HyClone "defined", Thermo Scientific, Logan, UT, USA, catalogue no. SH30070.03), 0.1 mM nonessential amino acids (Gibco-Brl, Invitrogen Ltd, Paisley, UK, catalogue no. 11140–050), 0.1 mM 2-mercaptoethanol (Sigma-Aldrich, St. Louis, MO, USA, catalogue no. M6250), and 1,000 U/ml ESGRO Leukemia Inhibitory Factor (LIF) (Millipore, Billerica, MA, USA, catalogue no. ESG1107), on gelatin-coated dishes. 2 × 10^6^ EBRTc cells were transfected with the PTHC-*Tbx1-myc* plasmid and with the pCAGGS-Cre vector, as described [17]. Thirty puromycin-resistant clones were selected and eleven of them were screened by Southern blotting. One positive clone was selected for further analysis and named mES-*Tbx1*Tet^OFF^. For embryoid body (EB)-based differentiation, cells were seeded in hanging drops (400 cells/drop) placed on the lids of tissue culture dishes and incubated for 2 days, and then cultured in suspension for 3 more days in Petri dishes. All steps were performed in complete DMEM medium, supplemented with 15% FBS, 7.5μg/ml tetracycline (Sigma, catalogue no. T7660) without LIF. 5-day-old EBs were plated on gelatin-coated plates for further analysis with or without tetracycline. For clonal analysis EBs were dissociated at day 4.75 using a kit (Miltenyi Biotec GmbH catalogue. n. 130-096-348) according to manufacturer’s instructions, VEGFR2+ cells were isolated using CD309 Miltenyi microbeads kit (Miltenyi Biotec GmbH catalogue. n. 130-090-858). VEGFR2+ cells were plated at clonal density (10^4^ cells/60mm plate) in media with LIF for 7 days. Individually isolated colonies were then picked, trypsinized and plated in individual 96 wells, grown for 3 days and then divided in two plates, Tet+ and Tet-. After 3 days in culture, RNA was extracted and processed for RT-PCR.

P19Cl6 cells [18] were cultured in alpha-MEM, 10% FBS and 1% Glutamine. For differentiation 5x10^5^ cells were plated on a 35-mm tissue culture dish. After one day, the medium was replaced with growth media containing 10μM 5-Azacytidine for 24 h. From the next day, cells were incubated in growth media containing 1.0% DMSO that was changed daily. Day 0 of differentiation is defined as the day of 5-Aza addition to the media.

For siRNA-mediated knockdown of *Tbx1*, cells were cultured in 35 mm dishes and transfected at day 0 with siRNA (LifeTecnology n. 4390771 s-74767; s-74769) using Lipofectamine-RNAi-MAX transfection reagent (LifeTechnology) according to the manufacturer protocol. Non-targeting siRNA was used for control transfections. Cells were harvested at day 1 for RNA extraction and reverse transcription.

### RNA Isolation

Total RNA was isolated with TRIZOL (Invitrogen) and reverse-transcribed using the High Capacity cDNA reverse transcription kit (Applied Biosystem catalogue. n. 4368814). Quantitative real-time PCR (qRT-PCR) was performed using SYBR Green PCR master mix (Applied Biosystem). Relative gene expression was evaluated using the 2^–ΔΔCt^ method, and *Gapdh* expression as normalizer. Primers are listed in S1 Table. Expression data are shown as the mean ± SEM. Statistical analysis was performed using the Student’s t-test for expression data, and chi-square test for clonal analysis.

### Immunohistochemistry and *In Situ* Hybridization

Animal research was conducted according to EU and Italian regulations. The animal protocol has been approved by the animal welfare committee of the Institute of Genetics and Biophysics (Organismo per il Benessere Animale or OPBA-IGB), protocol n.: 0002183 (June 4, 2013). *Tbx1* ^*+/-*^ mice [19] were crossed to obtain *Tbx1* ^*-/-*^ embryos. Genotyping was performed as in the original report. Embryos were fixed in 4% paraformaldeyde (PFA)/PBS at 4°C overnight and embedded in OCT. 10 μm sagittal and transverse sections were cut using a cryostat and were post-fixed in 4% PFA at room temperature for 5 min prior to immunostaining. Sections were briefly rinsed with PBS, treated with 0.5% H_2_O_2_ in ethanol to block endogenous peroxidase activity and blocked in 20% Goat Serum (GS) in PBS with 0.05% Tween for at least 30’ at room temperature, incubated in biotin anti mouse CD309 (VEGFR2)(Biolegend) overnight at 4°C in 5% GS in PBS, 0.05%Tween; the signal of biotin anti mouse CD309 antibody was enhanced using Vectastain Elite-ABC kit reaction (PK 6200, Vector Laboratories, Burlingame, CA) and visualized using DAB Peroxidase (HRP) Substrate Kit (SK-4100 Vector Laboratories, Burlingame, CA). For in situ hybridization, *Vegfr2* or *Tbx1* anti-sense RNA probes were labeled using the DIG-RNA labeling kit (Roche) and hybridized to cryosections following published methods [20]. Digital photographs were taken with a Leica DM6000 microscope and acquisition software LAS V4.1.

### Flow Cytometry and Immunofluorescence

EBs were dissociated into single cell suspension using Trypsin-EDTA solution. 1.5-2x10^6^/ml cells were collected and fixed for 30 min at 4°C in 70% cold EtOH (anti-NKX2.5 and anti-GATA4 antibodies), or 10 min at RT in 4%PFA (anti-P-H3 antibodies). Samples were permeabilized in 0.5%NP-40/PBS /1%BSA buffer for 10 min with slow shaking at RT, and incubated with the primary antibody (1:50 for the anti-NKX2.5 and anti-GATA4; 1:100 for the anti-P-H3) for 30 min at RT in 0.1%NP-40/PBS/1%BSA. After washing in 0.1%NP-40/PBS/1%BSA, samples were incubated with 1:500 anti-goat FITC or anti-mouse Texas Red for 15 min at RT. All washings were performed in PBS/1%BSA at RT. Flow cytometry with anti VEGFR2 antibodies was performed according to manufacturer's protocol (Biolegend). Immunofluorescence was performed on cells plated on gelatin-treated chamber-slides (Permanox, ThermoScientific) at day 5 of differentiation, and fixed at day 6 for anti-P-H3 and day 8 for VEGFR2. Antibodies used are listed in S1 Table.

## Results

### Generation of a Dynamic Model of *Tbx1* Gene Expression

**Fig 1:**
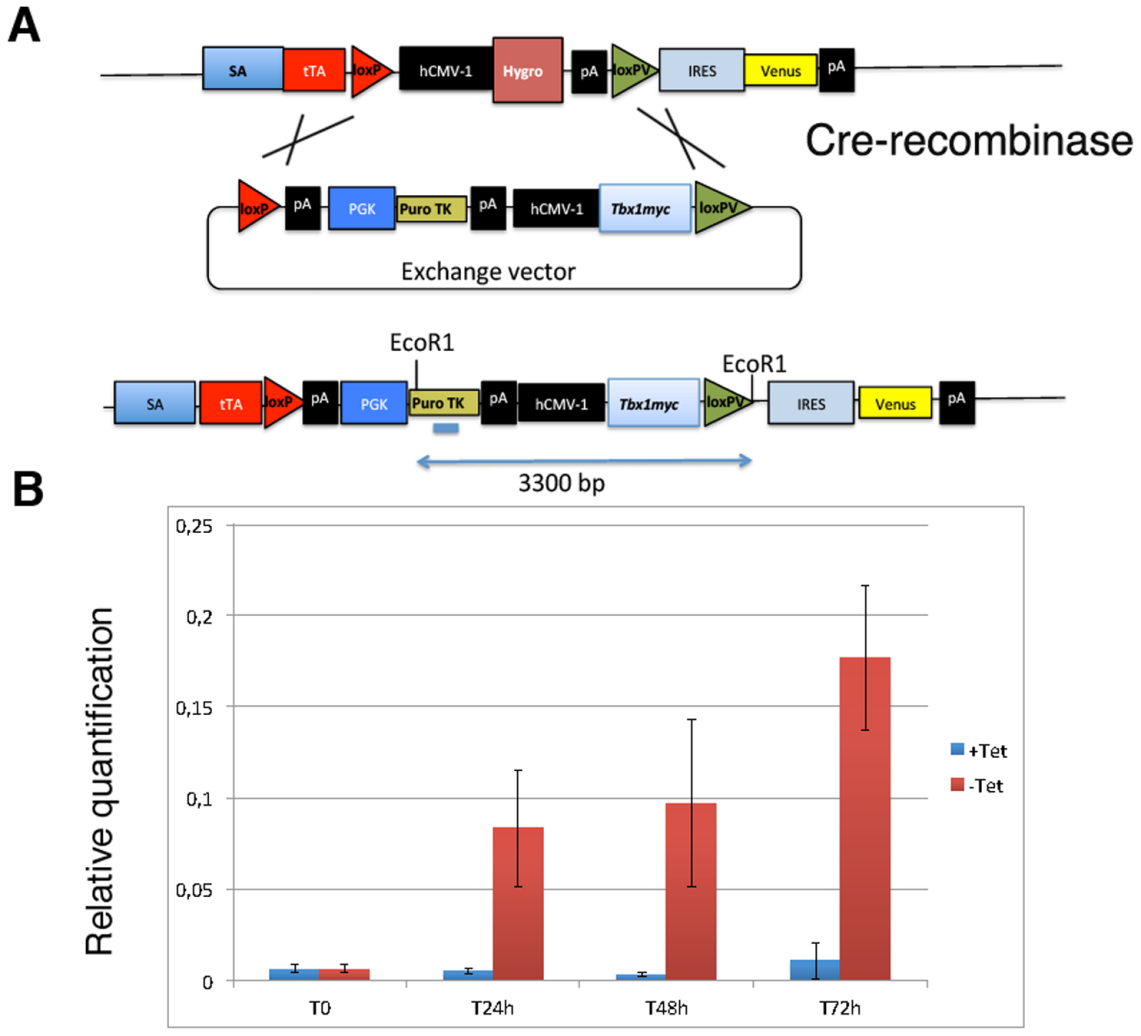
Generation of the ES cell line inducible system mES-*Tbx1*Tet^OFF^. A: *top*, docking site at the Rosa-Tet locus of the EBRTc cell line [17]; *middle*: exchange vector (PTHC-*Tbx1-myc*) carrying a *Tbx1* cDNA; *bottom*: recombinant allele after Cre-induced recombination. B: qRT-PCR analysis of *Tbx*1 expression in undifferentiated mES-*Tbx1*Tet^OFF^ cells. Values are relative to that at tetracycline removal time (0h) and are the average of three different experiments. Error bar: S.E.M.

We have inserted a *Tbx1* cDNA into the Rosa 26 locus of mouse embryonic stem cells EBRTc using a docking system that includes a Tet^Off^ regulation construct [17]. The strategy is illustrated in Fig 1A. The resulting engineered ES cell line is hereafter referred to as mES-*Tbx1*Tet^Off^. This cell line expresses *Tbx1* upon removal of tetracycline (Fig 1B; average of 3 different experiments). *Tbx1* RNA was detectable in undifferentiated cells 24 hrs after tetracycline removal from ES media (Fig 1B). Transgene *Tbx1* expression was also confirmed by using specific primers in the 3’-tagged region (S1 Fig). Both induced and not induced cells were able to differentiate into beating cells (S1 Video and S2 Video).

### TBX1 Enhances Cell Proliferation and Modulates the Expression of Cardiac Differentiation Markers

**Fig 2:**
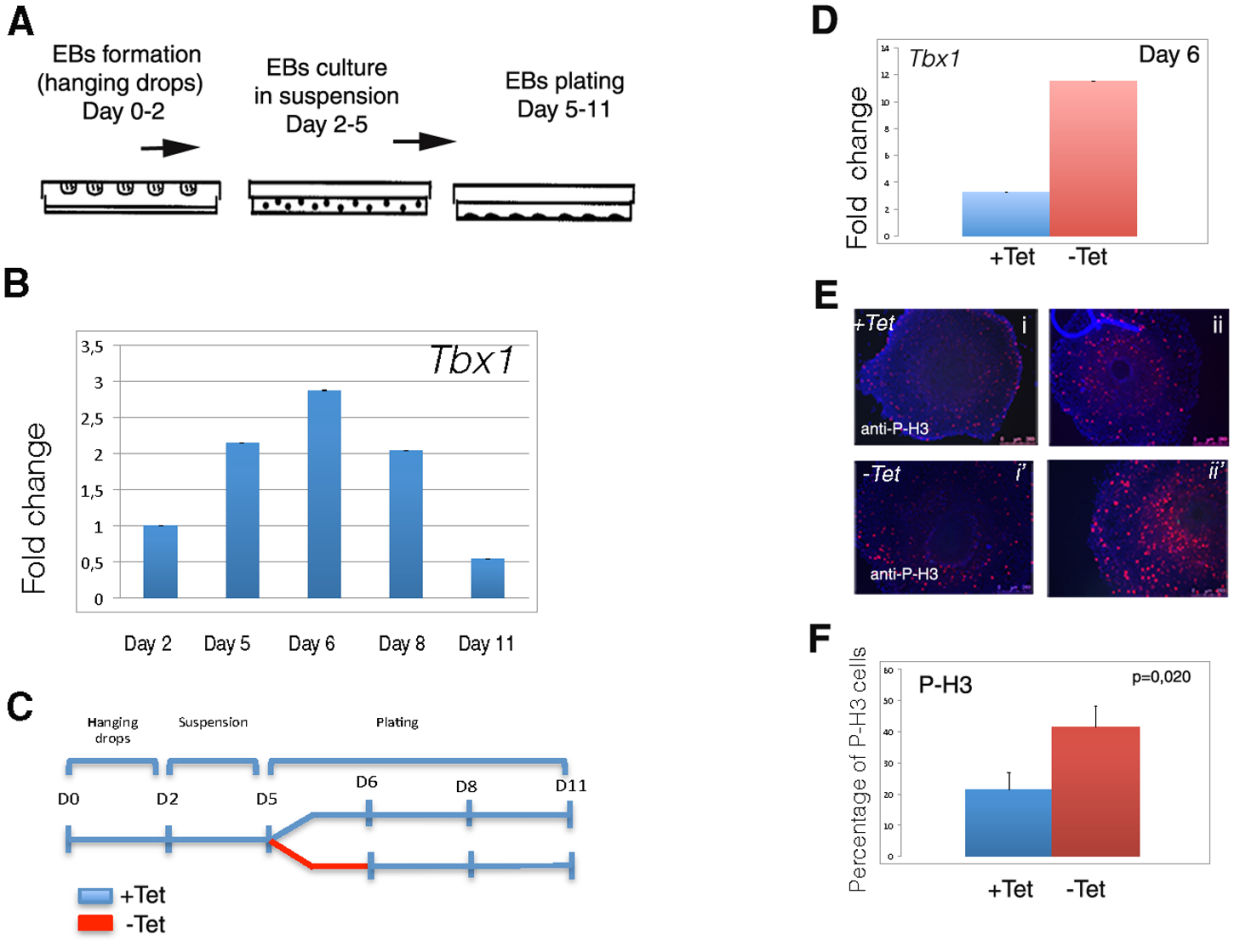
Differentiation assay of the mES-*Tbx1*Tet^OFF^ cell line. A: Scheme of experimental protocol. B: qRT-PCR assay of *Tbx1* expression in uninduced (+Tet) mES-*Tbx1*Tet ^OFF^ cells. A peak of expression is evident at day 6. C: Schematic representation of the experimental strategy used for pulse *Tbx1* expression. D: qRT-PCR analysis of *Tbx1* expression at day 6 after 24hrs without tetracycline. E: Immunofluorescence with an anti-P-H3 antibody on two colonies of mES-*Tbx1*Tet ^OFF^ cells with and without tetracycline at day 6. F: graphic representation of quantitative evaluation of mitotic activity using flow cytometry.

To test the effect of *Tbx1* expression in our ES cell system, we have used mES-*Tbx1*Tet^Off^ cells in the EB-based differentiation protocol illustrated in Fig 2A. In this system, endogenous *Tbx1* expression was detected at day 5 of differentiation, reached a peak at day 6 and then it decreased (Fig 2B). To enhance *Tbx1* expression, we removed tetracycline from the tissue culture media at day 5 for 24 hrs and then continued the differentiation protocol in the presence of tetracycline (Fig 2C). Under these conditions, *Tbx1* expression was enhanced by approximately 3 folds at day 6 (Fig 2D). The number of mitotic cells was quantified at day 6 using an antibody anti-P-H3 by immunofluorescence (Fig 2E) and by flow cytometry. Fig 2F shows the average of three experiments. The increased mitotic activity in cells subjected to *Tbx1* expression pulse is consistent with our previous data [9] and indicates that the protein encoded by the transgene is biologically active.

**Fig 3:**
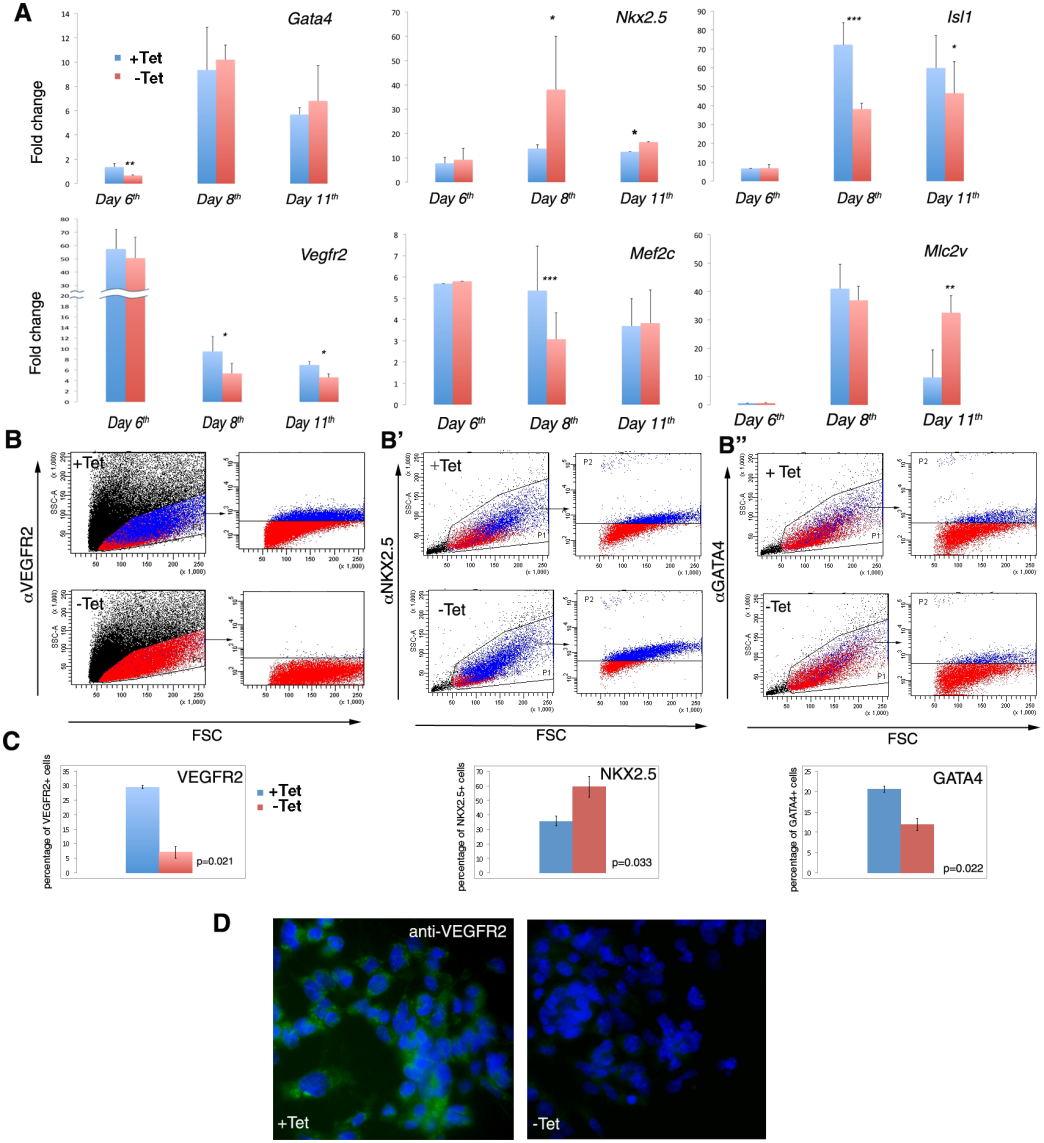
*Tbx1* expression pulse at day 5 produces persistent changes of expression of cardiac markers. A: qRT-PCR assay at Day 6, Day 8 and Day 11 of mES-*Tbx1*Tet ^OFF^ cells with (red) and without (blue) pulse at Day 5. The data are represented as mean of fold change relative to Day 5 value (not shown) of four different experiments. Error bars indicate SEM. (* = *p*-value<0.05; ** = *p*-value <0.005; *** = *p*-value <0.0005). B: Flow cytometric evaluation of the effects of *Tbx1* pulse on VEGFR2, NKX2.5 and GATA4 cell population at Day 8. Flow cytometry at Day 8 shows a decrease of the numbers of VEGFR2+ and GATA4+ cells (B, B”) and an increase of the number of NKX2.5+ cells (B’) after *Tbx1* pulse. C: The histograms show the mean percentage of positive cells in three different experiments. Error bar indicate SEM. D: Immunofluorescence detection of VEGFR2 with (Tet-) or without (Tet+) *Tbx1* pulse. FSC: Forward Scatter.

Next, we investigated the effects of increased *Tbx1* expression on cell differentiation. To this end, we tested the expression of cardiac and endothelial lineage differentiation markers *Nkx2*.*5*, *Isl1*, *Gata4*, *Vegfr2*, *Mef2c* and *Mlc2v* by quantitative real time reverse-transcription PCR (qRT-PCR) at days 6, 8, and 11, of differentiation using the scheme indicated in Fig 2C, with and without a 24hrs-pulse at day 5. At day 6 *Gata4* expression was down regulated in cells subjected to *Tbx1* pulse, while the other markers did not change significantly (Fig 3A). At day 8, *Nkx2*.*5* was up regulated while *Mef2c*, *Vegfr2* and *Isl1* were down regulated. At day 11, *Nkx2*.*5* and *Mlc2v* were up regulated while *Vegfr2* and *Isl1* were down regulated compared to controls without pulse (Fig 3A). At day 8, flow cytometry, confirmed and quantified the reduction of VEGFR2+ cells, and showed reduction of GATA4+ cells and increase of NKX2.5+ cells (Fig 3B and 3C). The reduction of VEGFR2 expression was also visualised by immunofluorescence, at the same day (Fig 3D). Thus, after a *Tbx1* expression pulse, differentiating ES cells have an "early" response (detected soon after the pulse at day 6) characterized by down-regulation of *Gata4*, and a "late" response (detectable after 48 hrs from the pulse, day 8) characterized by the expansion of the NKX2.5+ cell population and shrinkage of the VEGFR2+ and GATA4+ cell populations. We were intrigued by the suppressive effect of *Tbx1* over expression on *Vegfr2* expression. *Vegfr2* is a marker of EC progenitors and hematopoietic cells, but it also identifies a sub-population of cardiac progenitors [13,21,22]. Thus, we analysed the impact of *Tbx1* loss of function (by siRNA knock down) on *Vegfr2* expression in an independent cell differentiation model. S2A Fig shows the expression of *Tbx1* and *Vegfr2* during differentiation of P19Cl6 cells [18], note the nearly complementary pattern. We knocked down *Tbx1* expression at day 0 and found that at day 1 the expression of *Vegfr2* was significantly increased in 3 repeated experiments (S2B' Fig), confirming that *Tbx1* is a negative regulator of *Vegfr2*.

### VEGFR2+ Cells Express the Cardiac Marker *Smarcd3* in Response to *Tbx1* Expression

**Fig 4:**
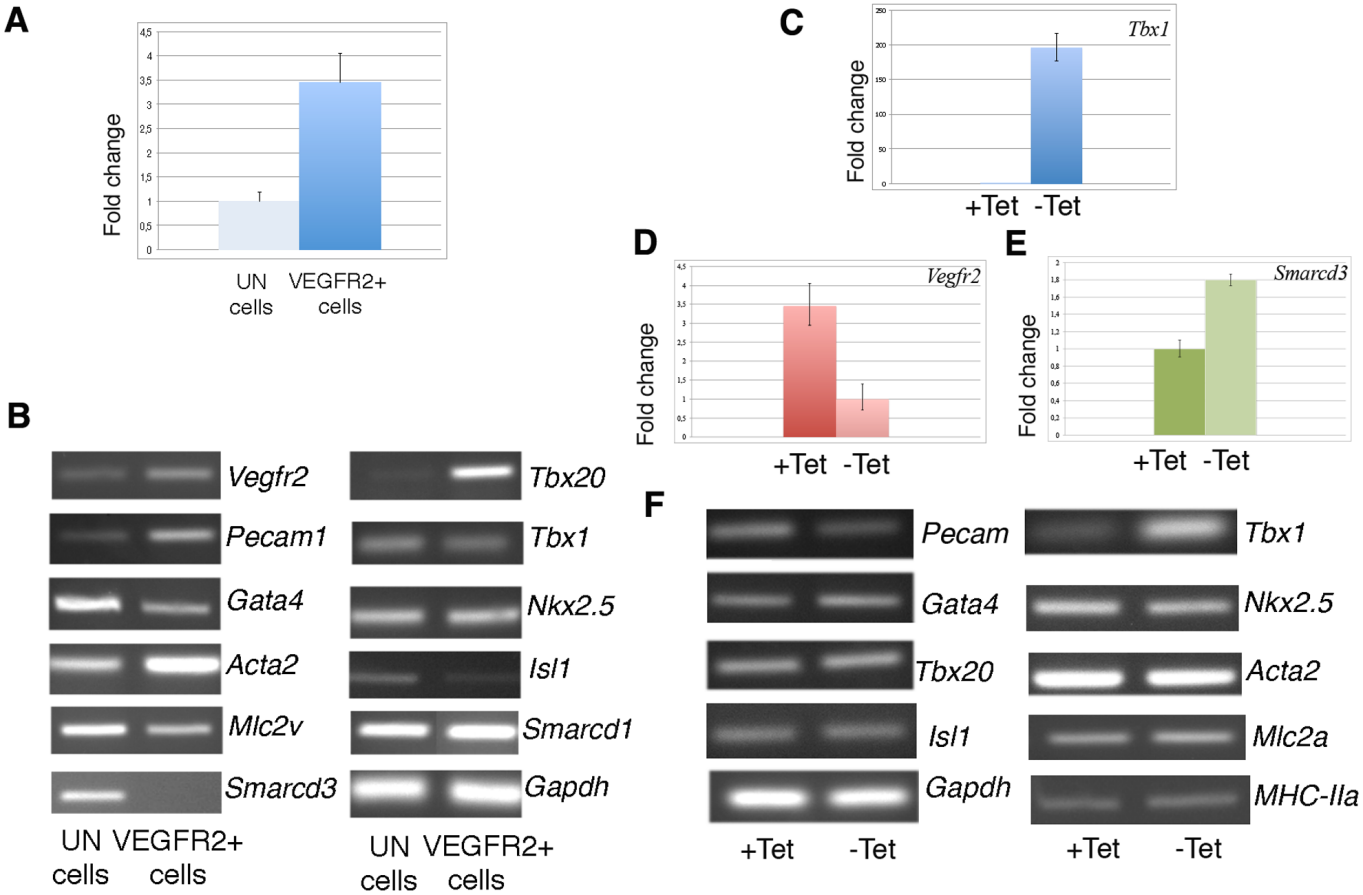
Expression profile of VEGFR2+ cells isolated at Day 4.75 of differentiation from mES-*Tbx1*Tet^Off^ cells and subjected to additional 48hrs in culture, with or without tetracycline. A: qRT-PCR assay of *Vegfr2* expression of cells bound to magnetic beads (VEGFR2+ cells) and unbound (UN). B: RT-PCR analysis of VEGFR2+ and unbound cells. C: qRT-PCR of *Tbx1* expression in VEGFR2+ cells. D,E: qRT-PCR evaluation of *Vegfr2* and *Smarcd3* expression in VEGFR2+ cells with and without Tet.

We have followed up the relationship between *Tbx1* and *Vegfr2* and explored the destiny of VEGFR2+ cells following transient expression of *Tbx1*, we have subjected mES-*Tbx1*Tet^Off^ cells to the differentiation protocol shown in Fig 2C, but cells were harvested at day 4.75 (as in [12]). At this time of differentiation, the percentage of VEGFR2+ cells was about 15% of total as assayed by flow cytometry (S3 Fig). Then we isolated VEGFR2+ cells using magnetic beads, we tested markers of cardiac and endothelial lineages in the VEGFR2-enriched cell population, and compared their expression with that of the unbound cell population (Fig 4A and 4B). VEGFR2+ cells were positive for all the markers tested (including *Tbx1*), with the exception of *Smarcd3*, a muscle differentiation marker (Fig 4B). In contrast, the unbound population expressed *Smarcd3* but did not express *Tbx20* (Fig 4B). Next, we performed another experiment using the same scheme, but the VEGFR2-enriched cell population was split in two samples, one of which was subjected to *Tbx1* induction by withdrawing tetracycline for 24 hrs. After 24hrs of induction, we extracted RNA and performed marker analyses. Results revealed changes of *Vegfr2* and *Smarcd3* expression (Fig 4D and 4E) but the other markers showed a similar level of expression as tested by standard RT-PCR, (Fig 4F). These results suggest that *Tbx1* expression in VEGFR2+ cells from differentiating ESCs may confer or reinforce a muscle differentiation potential.

### Clonal Analysis: VEGFR2+ Cells Are Heterogeneous, Plastic, and Their Fate Can Be Modulated by *Tbx1* Expression in Tissue Culture

**Fig 5:**
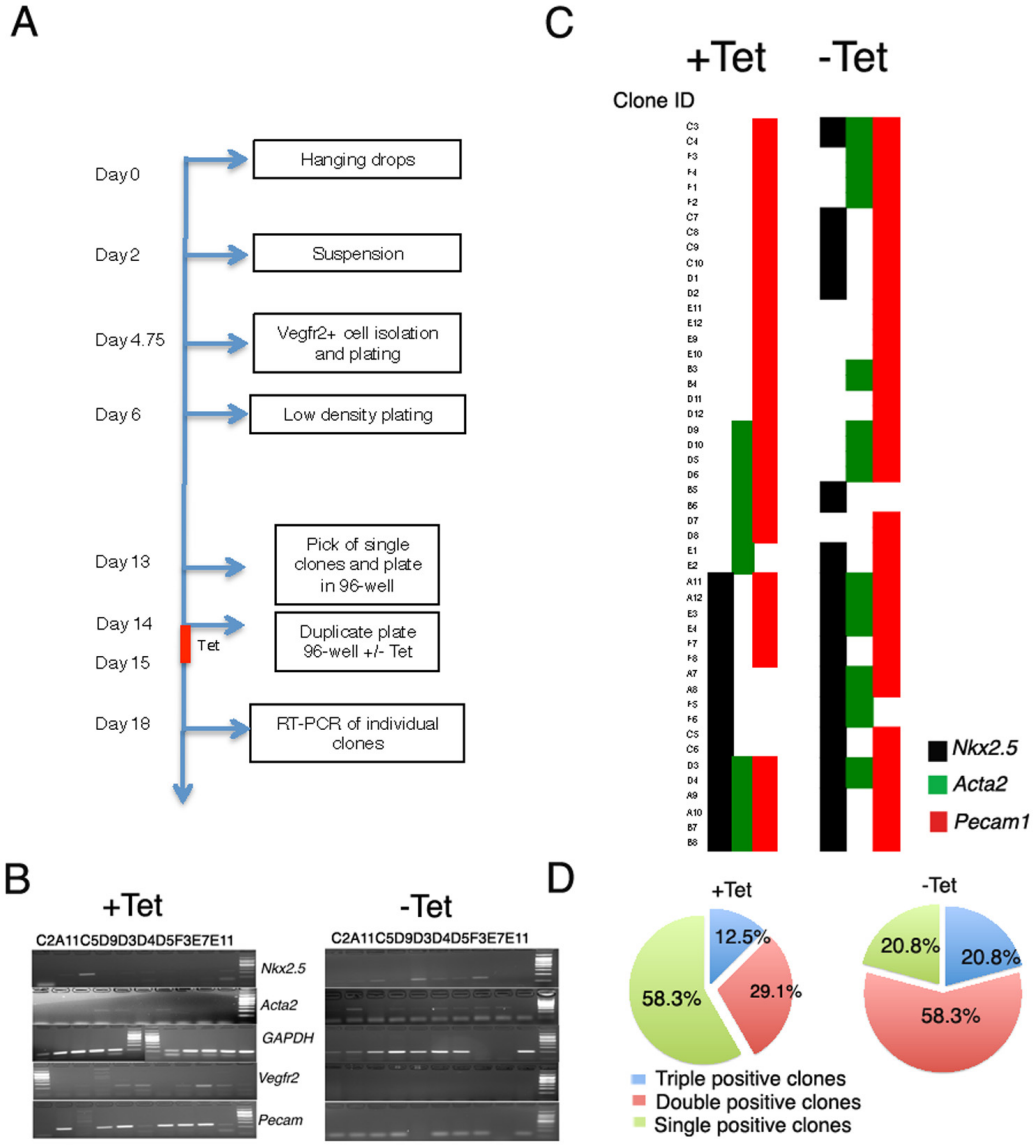
Clonal analysis of VEGFR2+ cells. A: schematic representation of experimental strategy. B: Examples of RT-PCR analysis on VEGFR2+ cells in absence and in presence of *Tbx1* pulse. C: Color map panels of marker positivity of individual clones with or without *Tbx1* pulse. D: Graphic representation of clone multiplicity, based on the number of positive markers with and without tetracycline. Blue represents the percentage of clones that were positive for all three markers; Red represents the percentage of clones positive for two markers; Green represents the percentage of clones that are positive for one marker.

To explore further the ability of TBX1 to “push”VEGFR2+ cells toward a cardiac fate, we performed clonal analysis. The experimental procedure is outlined in Fig 5A. Briefly, at day 5 of differentiation, VEGFR2+ cells were isolated and plated at clonal density. They were grown in the presence of LIF and after 7 days we picked individual, well isolated colonies, and seeded them in 96-well plates. After one day, plates were duplicated and cultured with and without tetracycline for 24 hrs. After additional 3 days of culture in the absence of LIF we harvested cells and extracted RNA to assay expression by RT-PCR. *Vegfr2* expression was retained in 58.3% (n = 28) of control clones tested (n = 48) and in 12.5% (n = 6) of clones subjected to *Tbx1* pulse. Next, we tested the expression of cardiac lineage markers *Nkx2*.*5*, *Pecam1*, and *Acta2* indicative of cardiomyocyte precursors, EC and smooth muscle cells, respectively (examples of raw data are shown in Fig 5B). A summary of results is shown schematically in Fig 5C, which includes only the clones positive for at least one of those markers (n = 48). In the absence of *Tbx1* pulse, 41.6% of clones (n = 20) expressed only the endothelial marker *Pecam1*. 12.5% of clones (n = 6) expressed two markers, *Nkx2*.*5* and *Pecam1* and 12.5% of clones (n = 6) expressed all three marker. These results indicate that under the conditions tested, the most prevalent profile is endothelial, while a relatively low percentage of clones shows a cardiac progenitor profile (triple positive clones).

In contrast, the same clones, after *Tbx1* pulse, exhibited a more diverse pattern (Fig 5C, right panel). In particular, only 16.6% of clones (n = 8) expressed *Pecam1* alone, and about 33% of clones (n = 16) were double positive for *Nkx2*.*5* and *Pecam1*, and 20.8% of clones (n = 10) was triple positive (Fig 5C). The overall number of *Pecam1+* clones (independently from the expression of other markers) did not change significantly with the *Tbx1* pulse (40 *vs*. 44), thus *Tbx1* is not a *Pecam1* gene suppressor. The most evident change is the multiplicity of markers expressed by clones that have undergone *Tbx1* pulse (Fig 5D). Indeed, the percentage of clones positive for at least two markers was 41.6% (n = 20) without *Tbx1* pulse, but increased to 79.1% (n = 38) with induction (p = 0.000172; chi square-test). The increased multiplicity is due to an increase of the number of clones positive for *Nkx2*.*5* (from 18 to 30; p = 0.0143) and to a reduction of the number of clones expressing only *Pecam1* (from 20 to 8; p = 0.0070).

These data suggest that forced *Tbx1* expression increases the likelihood of expression of multiple cardiac markers in clones derived from VEGFR2+ cells.

### *Vegfr2* Is Expressed in the Posterior Second Heart Field (pSHF) and Its Expression Domain Is Expanded in *Tbx1* ^-/-^ Mouse Mutants

**Fig 6:**
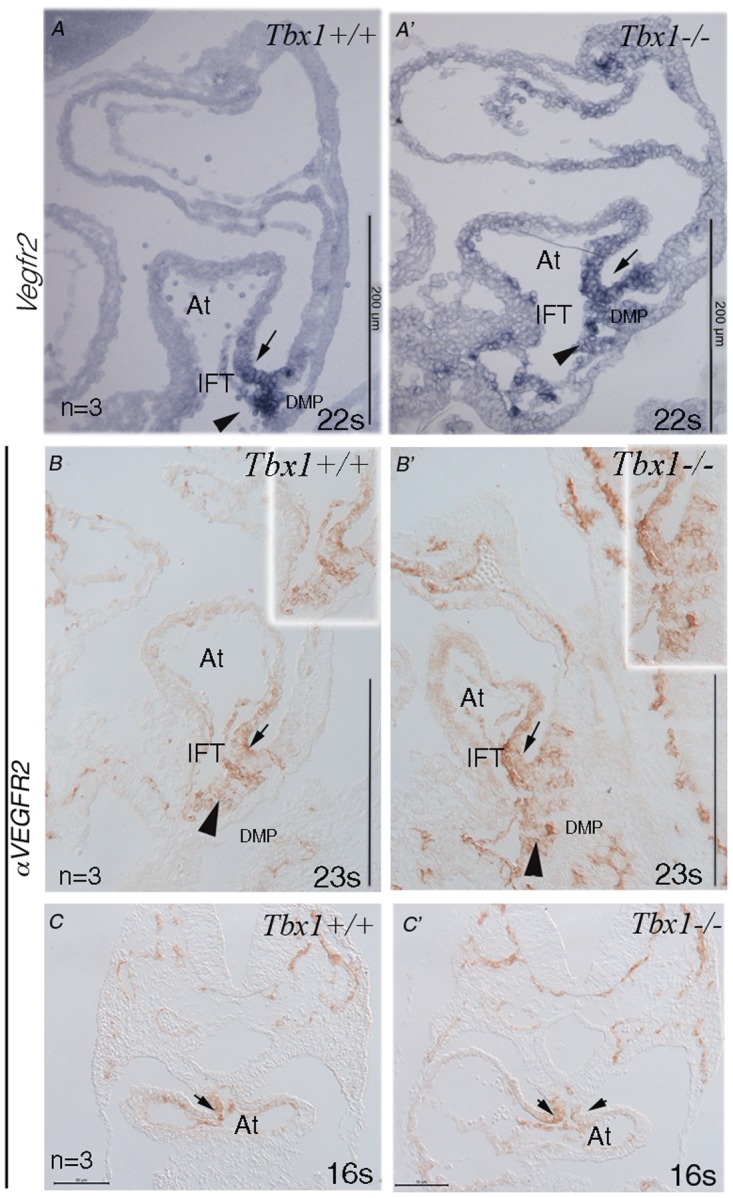
*Vegfr2* expression in wild type and *Tbx1* ^-/-^ mouse embryos E9.5 and E9.0. A, A’: In situ hybridization of wild type and *Tbx1* ^-/-^ embryos. B-C': Immunohistochemisty with an anti-VEGFR2 antibody on sagittal and transverse sections. The arrows indicate the expression domain in the pSHF. The arrowheads indicate the DMP region (n = number of embryos examined). Scale bars: 200 μm in A, A’, B, B’. 50 μm in C, C’. s: number of somites.

**Fig 7:**
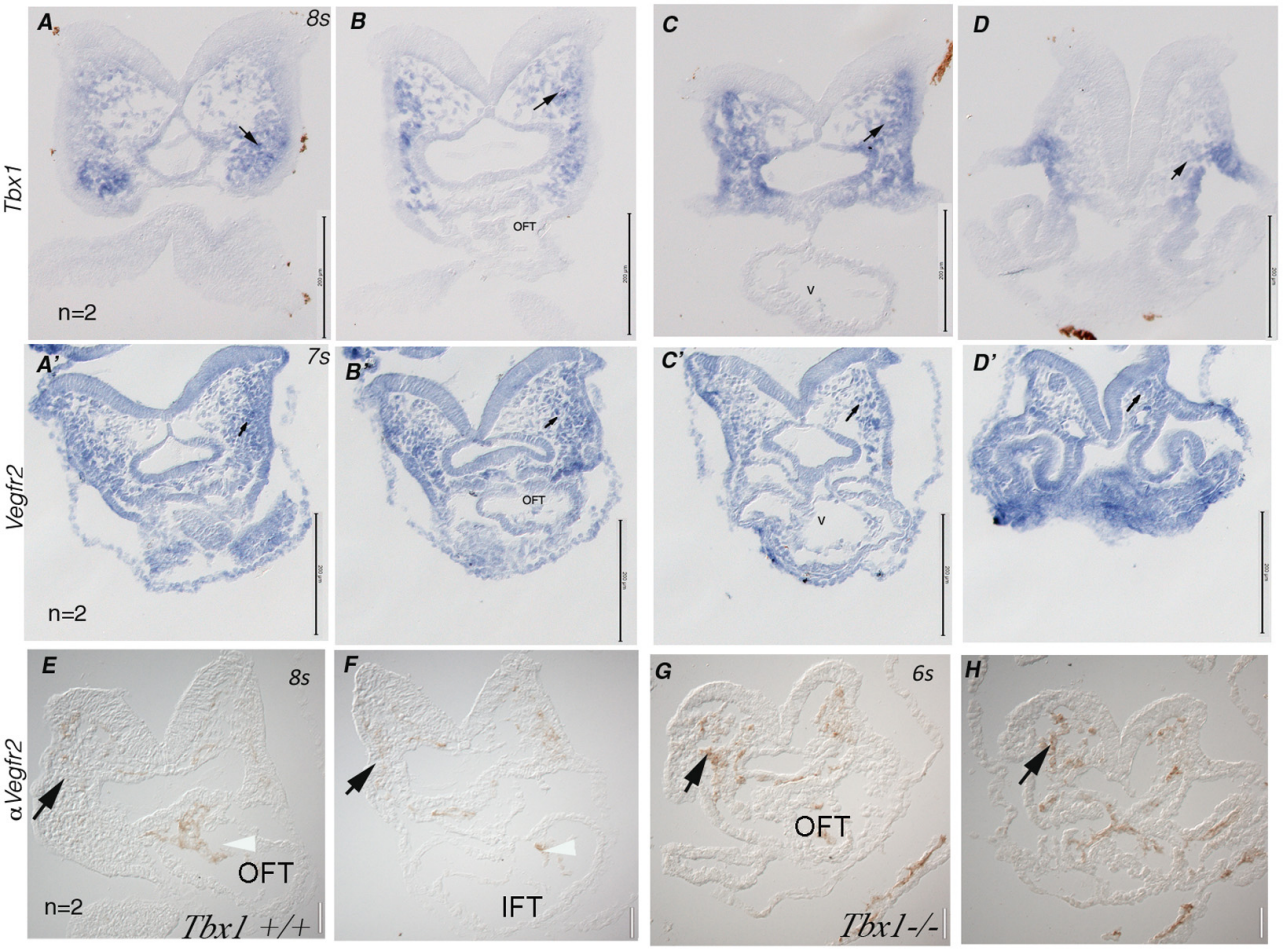
*Vegfr2* and *Tbx1* expression in E8.5 mouse embryos. A-D': In situ hybridization of *Tbx1* (A-D) or *Vegfr2* (A'-D') shown in a rostro-caudal series of transverse sections of wild type embryos. Arrows indicate *Vegfr2* expression in the pharyngeal mesenchyme. E-F: VEGFR2 immunohistochemistry in similar sections as in A and B. G-H: VEGFR2 immunohistochemistry in corresponding sections of a *Tbx1* ^*-/-*^ embryo. Arrows indicate VEGFR2+ cells in the pharyngeal mesenchyme. OFT: cardiac outflow tract. IFT: cardiac inflow tract. Scale bars: 200 μm in A-D, A’-D’. 50 μm in E-H. s: number of somites.

To validate our *in vitro* findings, we tested whether TBX1 has an effect on *Vegfr2* expression *in vivo*. At E9.5, *Vegfr2* is mostly expressed in EC but we found that it is also expressed in the mesodermal/epithelial-like tissue of the pSHF (arrows in Fig 6A and 6B) in the proximity of the IFT and in the dorsal mesenchyme protrusion region (DMP) (arrowheads in Fig 6A and 6B). In *Tbx1* ^-/-^ embryos, the *Vegfr2* gene or protein expression domains of the pSHF and DMP region expanded more dorsally and anteriorly (Fig 6A, 6A', 6B and 6B', examples from a total of 3 embryos analyzed). Moreover, we observed that in transverse sections, VEGFR2 immunostaining was asymmetric in WT embryos, but was symmetric in *Tbx1* ^-/-^ embryos (Fig 6C-C'). The expression of *Vegfr2* in the pSHF has not been reported to date. *Tbx1* is not expressed in the pSHF in the inflow region (S4 Fig), while *Vegfr2* is detected in this region at E8.5–9.0 (Fig 6). Therefore, we have analysed the expression patterns of *Vegfr2* and *Tbx1* at an earlier embryonic stage. At E8.5 (8 somites) *Tbx1* was strongly expressed in the pharyngeal mesoderm while *Vegfr2* was also expressed in the pharyngeal mesoderm but to a lesser extent (arrows in Fig 7A' and 7D'). Immunohistochemistry at the same developmental stage confirmed the presence of the VEGFR2 protein in the pharyngeal mesoderm (Fig 7E and 7F) and showed the expansion of this expression domain in *Tbx1* ^*-/-*^ embryos (Fig 7E and 7F'). Immunostaining also revealed that at E9.5, VEGFR2 expression in the I^st^ pharyngeal arch is expanded in *Tbx1* ^-/-^ embryos, compared to wild type (S5 Fig).

Overall, our data show that TBX1 is a negative regulator of VEGFR2 expression during mouse embryogenesis in specific tissues.

## Discussion

TBX1 plays a key role in the control of proliferation and differentiation of cardiac progenitor cells of the SHF, and in tissue culture it marks cells with cardiomyocyte, smooth muscle and endothelial differentiation potential [9]. In this work we provide the first evidence that TBX1 suppresses (directly or indirectly) the expression of the *Vegfr2* gene in differentiating ES cells and during embryonic development. How does TBX1 regulate *Vegfr2* gene expression? TBX1 may directly bind the *Vegfr2* gene. Indeed, computational analyses using the TRANSFAC database identified over 30 potential T-box binding sites within a 50Kb DNA segment centered on the gene (data not shown). It is also possible that the regulation is indirect. Indeed, our time-course expression analyses after *Tbx1* induction (Fig 3) showed that the expression of *Gata4* is down regulated earlier than that of *Vegfr2*, and it has been shown that *Gata4* regulates positively the *Vegfr2* gene [23], thus it is possible that the regulation of *Vegfr2* is mediated by *Gata4* regulation. It has been shown that *Gata4* expression is up regulated in *Tbx1* ^*-/-*^ embryos [24] and is down regulated in *Tbx1* overexpressing embryos [25]. *Vegfr2* is expressed in hematopoietic, endothelial and cardiac progenitors [12,26]. Conditional deletion of mouse *Vegfr2* in the endothelium (using an endothelial-specific Cre driver) causes embryonic lethality at E9.5-E10.5 associated with loss of the endocardium and hypoplasia of the OFT and RV [14]. This suggests that VEGFR2 (or the endocardium) has a non-autonomous role in the development of SHF-derived structures. A more recent study [27] has demonstrated that endothelial cells signal to cardiac neural crest-derived cells to support their migration. However, we found expression of *Vegfr2* in the pSHF (but not in the anterior SHF) as late as E9.5 and in the pharyngeal mesoderm at earlier stages. In addition, we found *Vegfr2* expression in the DMP, an SHF derivative that gives rise to the atrioventricular mesenchymal complex [28]. These expression data suggest that VEGFR2 may have a role in the SHF development independent from its role in the endocardium. Indeed, conditional deletion of *Vegfr2* in the *Isl1* expression domain, which includes the SHF, causes embryo lethality at E14.5 associated with cardiac defects but with preserved endocardium [14].

It has been shown that the pSHF and DMP of *Tbx1* ^-/-^ embryos have altered expression profile, and the DMP is hypoplastic, which may be the cause of the atrioventricular septal defects (AVSDs) found in these mutants [11]. Whether the expansion of the VEGFR2 domain in the pSHF and DMP of *Tbx1* ^-/-^ embryos could lead to AVSD is unknown because there is no information as to the phenotypic consequences of excessive VEGFR2 expression. However, in quail, increased VEGF signalling causes endocardial cell hyperplasia in the dorsal mesocardium region and affects heart development [29]. Future studies using transgenic mice should address whether or not VEGFR2 expansion contributes to the *Tbx1* mutant phenotype.

Is there a specific role of TBX1 in the fate of VEGFR2-expressing cells? Our tissue culture experiments using differentiating ES cells suggest that TBX1 works as a suppressor of *Vegfr2* expression. Overexpression of *Tbx1* in isolated VEGFR2+ cells also caused up regulation of *Smarcd3* expression, a marker of cardiac and skeletal muscle differentiation. Together these two findings suggest that TBX1 may favour cardiac fate at the expense of endothelial fate. However, clonal analysis of VEGFR2+ cells provided a more complex picture. Indeed, in basal conditions, clones derived from VEGFR2+ cells showed a prevalent endothelial fate. Transient induction of *Tbx1* expression did not suppress the endothelial marker gene *Pecam1* but was sufficient to activate, in many clones, *Nkx2*.*5* gene expression. Although some of the transcriptional changes of clones might be secondary to genetic drift, the overall trend is significant. These results could be interpreted in at least two different ways: A) *Tbx1* acts as an anti-differentiation factor and its overexpression tends to preserve a progenitor status; B) TBX1 acts as a “simple” activator of *Nkx2*.*5* gene expression. The latter interpretation, however, is consistent with previous findings showing down regulation of *Nkx2*.*5* in *Tbx1* ^-/-^ mutant [24]. The mechanism by which *Tbx1* may maintain a progenitor state is not clear. One possibility is that TBX1 induces a permissive chromatin state by interacting with the BAF/ SWI-SNF chromatin remodelling complex [30] and H3 lysine 4 methyltrasferase enzymes [30,31]. Another possibility is that the BMP-suppressive activity of TBX1, mediated by interaction with SMAD1 [32], may result in an anti-differentiation activity, as suggested by a study of self renewal of skin stem cells [33]. Our work identifies VEGFR2+ cells as a model for studying the plasticity of cardiac progenitors and for the identification of molecular players that maintain their progenitor status. We propose that TBX1 is a candidate for this function.

## Supporting Information

S1 Fig Quantitative real time PCR to evaluate transgene expression after *Tbx1* induction for 24 hrs, 48hrs and 72hrs. (TIF)

S2 Fig *Tbx1* knock down in P19Cl6 induces upregulation of *Vegfr2* expression. A-B: qRT-assay of *Tbx1* and *Vegfr2* expression during P19Cl6 differentiation. Note that *Tbx1* expression peaks at day 1 (A) while *Vegfr2* expression increases at day 2 (B). qRT-PCR assays of *Tbx1* (A') and *Vegfr2* (B') expression at day 1 after knock down of *Tbx1* at day 0.
(TIF)

S3 Fig VEGFR2+ cell quantification in differentiating mES-*Tbx1*Tet^OFF^ cells. by FACS analysis at 4.75 day of differentiation. (TIF)

S4 Fig *Tbx1* expression pattern in wild type embryos. In situ hybridizations of *Tbx1* are shown in two consecutive transverse sections near the venous pole of the heart, the arrows indicate the pSHF region. Wild type, 16 somite embryo (approx. E9.0). Scale bars: 50 μm s: number of somites. IFT: inflow tract; Ph: pharynx.
(TIF)

S5 Fig VEGFR2 expression in wild type and *Tbx1*-/- embryos. Immunoistochemistry of VEGFR2 shows expansion of expression in *Tbx1* null embryos. The panels show rostral, medial and caudal transverse sections of the pharyngeal region of wild type and *Tbx1* ^-/-^ embryos at three different somite stages. Scale bars: 50 μm. s: number of somites.
(TIF)

S1 Table List of primers and antibodies used. (XLSX)

S1 Video Differentiated mES-*Tbx1*Tet^OFF^ cell. The colonies showing pulsing activity without TBX1 induction (+Tet).
(AVI)

S2 Video Differentiated mES-*Tbx1*Tet^OFF^ cell. The colonies showing pulsing activity with TBX1 induction (-Tet).
(AVI)
