# Supplementary figures and images for: TBX1 Represses Vegfr2 Gene Expression and Enhances the Cardiac Fate of VEGFR2+ Cells
Source: PLoS One. 2015 Sep 18;10(9):e0138525. doi: 10.1371/journal.pone.0138525 (PMC4575176; doi:10.1371/journal.pone.0138525)

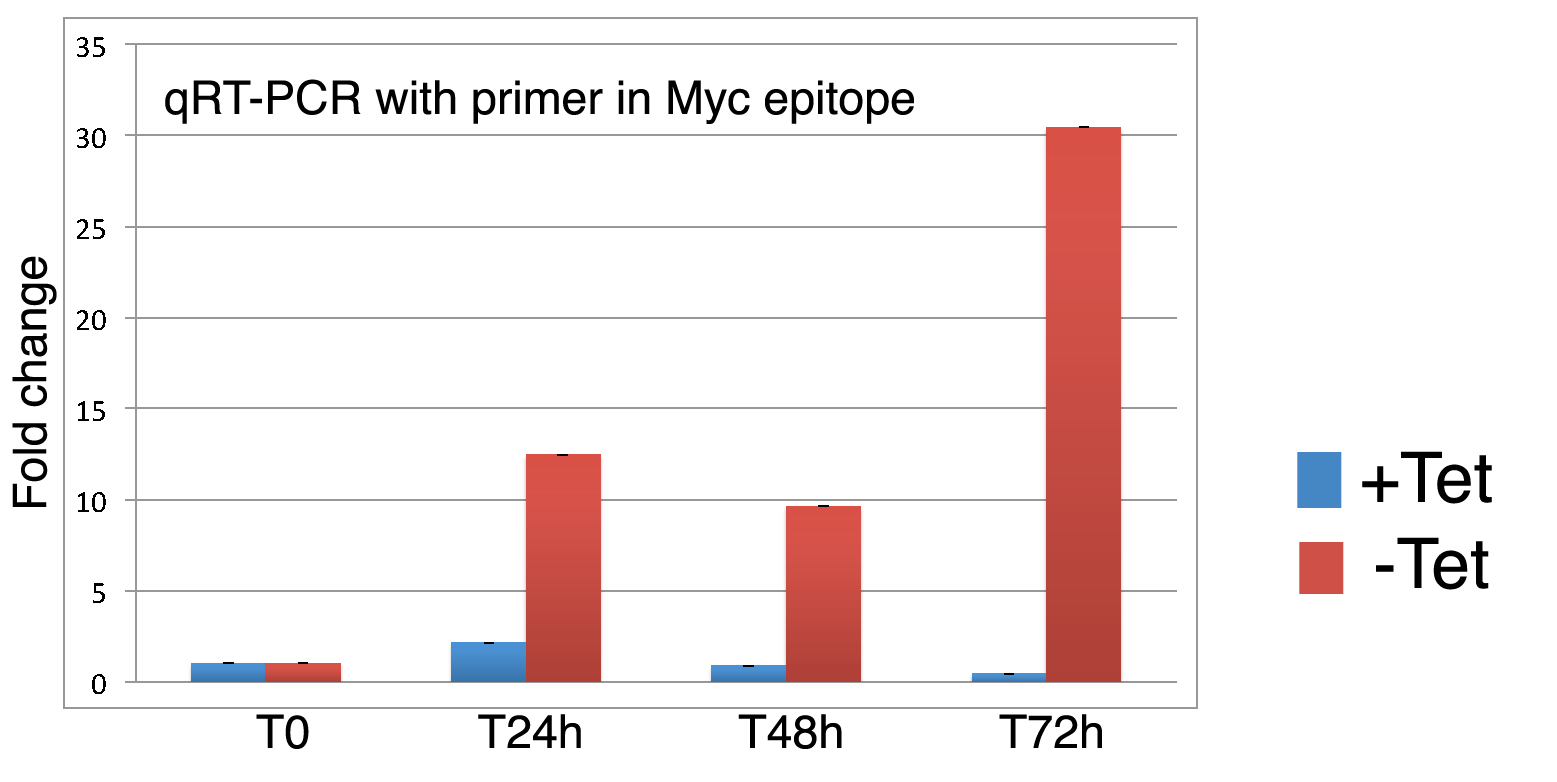

Supplement: S1 Fig — (TIF) [file pone.0138525.s001.tif]

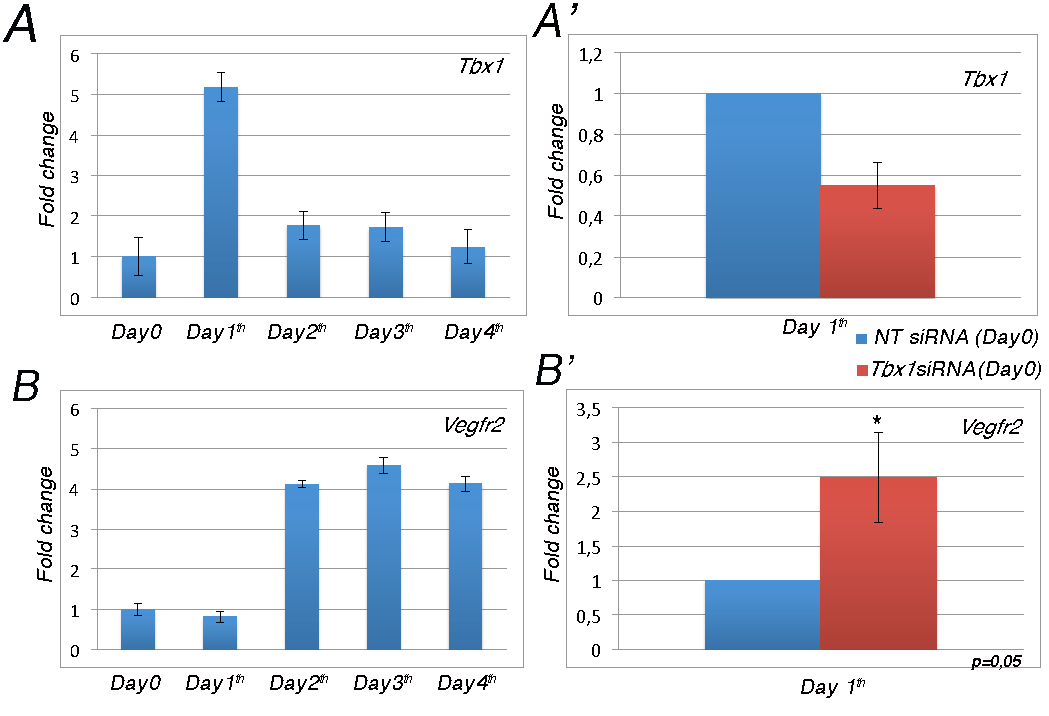

Supplement: S2 Fig — A-B: qRT-assay of Tbx1 and Vegfr2 expression during P19Cl6 differentiation. Note that Tbx1 expression peaks at day 1 (A) while Vegfr2 expression increases at day 2 (B). qRT-PCR assays of Tbx1 (A') and Vegfr2 (B') expression at day 1 after knock down of Tbx1 at day 0. (TIF) [file pone.0138525.s002.tif]

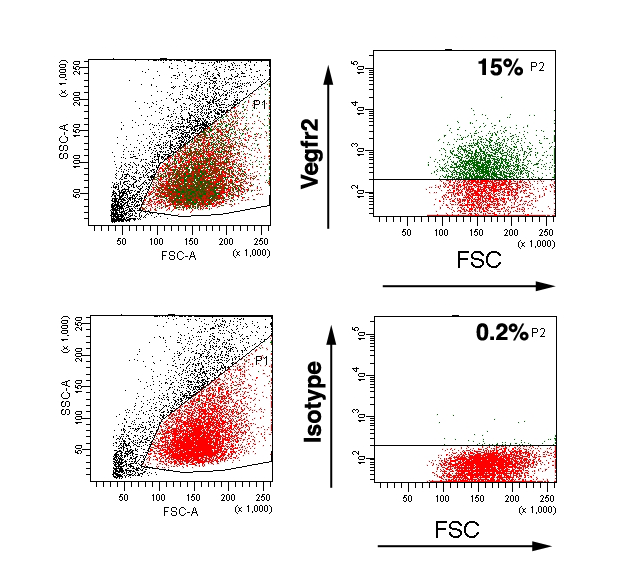

Supplement: S3 Fig — (TIF) [file pone.0138525.s003.tif]

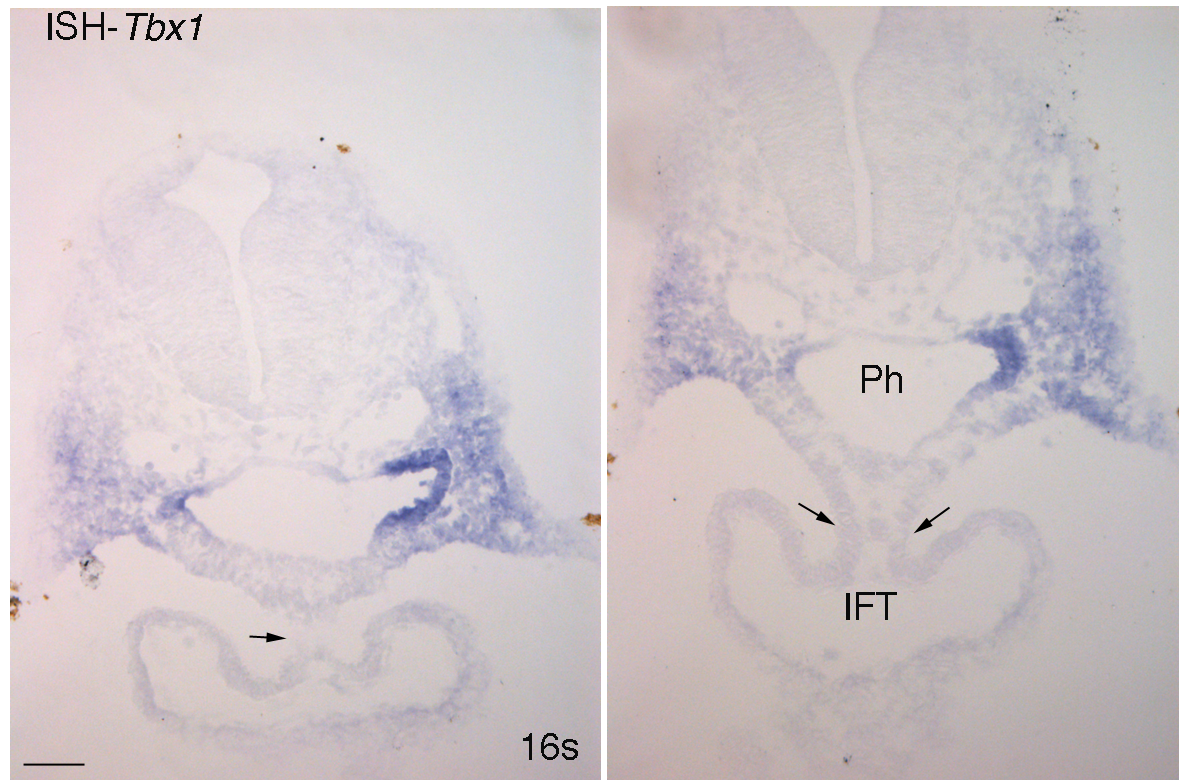

Supplement: S4 Fig — In situ hybridizations of Tbx1 are shown in two consecutive transverse sections near the venous pole of the heart, the arrows indicate the pSHF region. Wild type, 16 somite embryo (approx. E9.0). Scale bars: 50 μm s: number of somites. IFT: inflow tract; Ph: pharynx. (TIF) [file pone.0138525.s004.tif]

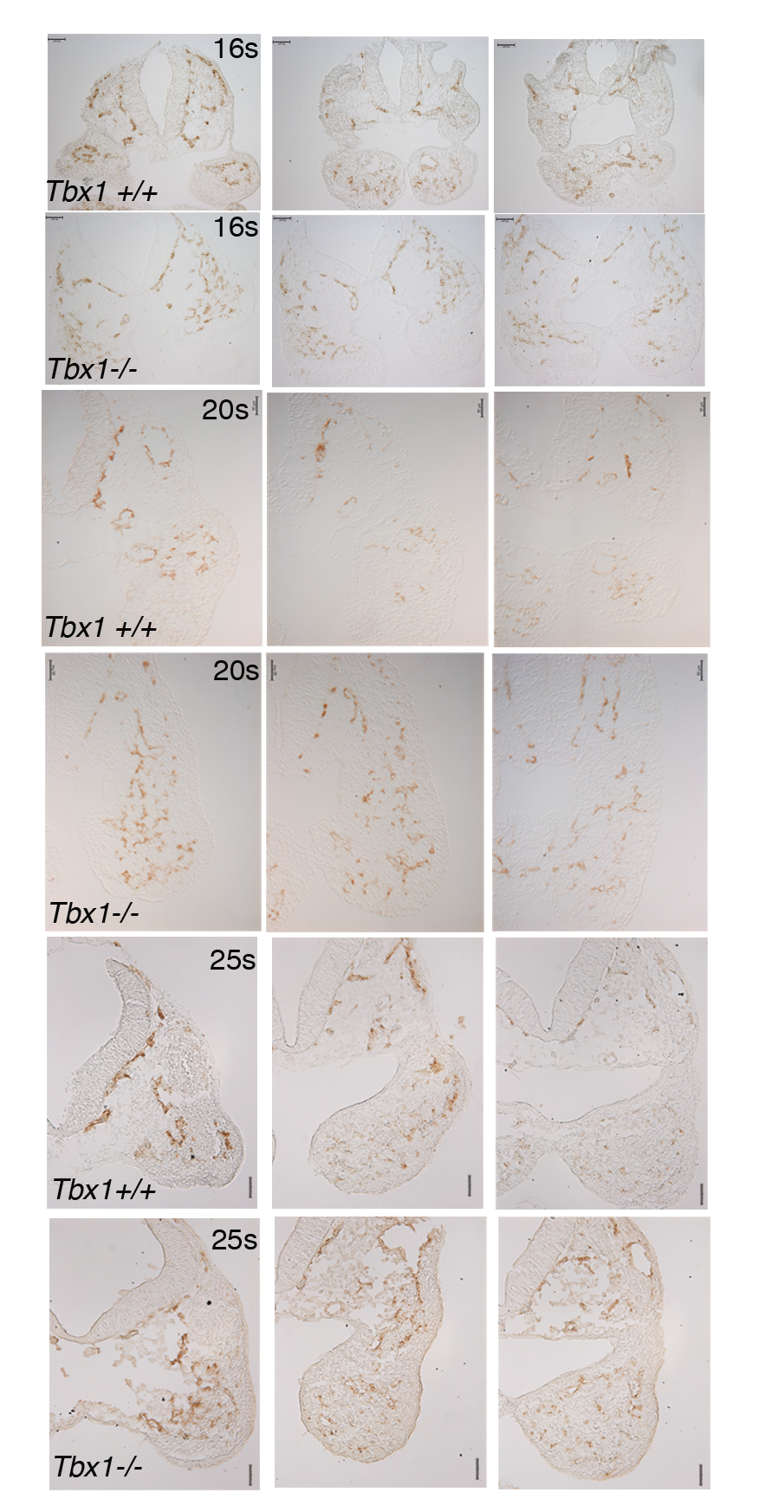

Supplement: S5 Fig — Immunoistochemistry of VEGFR2 shows expansion of expression in Tbx1 null embryos. The panels show rostral, medial and caudal transverse sections of the pharyngeal region of wild type and Tbx1 -/- embryos at three different somite stages. Scale bars: 50 μm. s: number of somites. (TIF) [file pone.0138525.s005.tif]
